# The impact of H/D exchange on the thermal and structural properties as well as high-pressure relaxation dynamics of melatonin

**DOI:** 10.1038/s41598-022-18478-0

**Published:** 2022-08-22

**Authors:** Paulina Jesionek, Barbara Hachuła, Dawid Heczko, Karolina Jurkiewicz, Magdalena Tarnacka, Maciej Zubko, Marian Paluch, Kamil Kamiński, Ewa Kamińska

**Affiliations:** 1 Institute of Chemistry, Faculty of Science and Technology, University of Silesia in Katowice, 40-007 Katowice, Poland; 2 Department of Pharmacognosy and Phytochemistry, Faculty of Pharmaceutical Sciences in Sosnowiec, Medical University of Silesia in Katowice, 41-200 Sosnowiec, Poland; 3 Institute of Physics, Faculty of Science and Technology, University of Silesia in Katowice, 41-500 Chorzow, Poland; 4 Institute of Materials Engineering, Faculty of Science and Technology, University of Silesia in Katowice, 41-500 Chorzow, Poland; 5 Department of Physics, Faculty of Science, University of Hradec Králové, 500 03 Hradec Králové, Czech Republic

**Keywords:** Medicinal chemistry, Physical chemistry, Medicinal chemistry, Pharmaceutics

## Abstract

In this paper, thermal properties, atomic-scale structure, and molecular dynamics (at ambient and high pressure) of native melatonin (MLT) and its partially-deuterated derivative (MLT-d_2_) have been investigated. Based on infrared spectroscopy, it was shown that treating MLT with D_2_O causes the replacement of hydrogen atoms attached to the nitrogen by deuterium. The degree of such substitution was very high (> 99%) and the deuterated sample remained stable after exposure to the air as well as during the melting and vitrification processes. Further calorimetric studies revealed the appearance of a peculiar thermal event before the melting of crystalline MLT-d_2_, which was assigned by the X-ray diffraction to a local negative thermal expansion of the unit cell. Finally, the high-pressure dielectric experiments indicated a few interesting findings, including the variation in the shape of the structural relaxation peak during compression, the difference in the pressure evolution of the glass transition temperature, and the temperature dependence of activation volume for both MLT species. The variations in these parameters manifest a different impact of the compression/densification on the dynamics of hydrogen and deuterium bonds in the native and partially-deuterated MLT, respectively.

## Introduction

Hydrogen bonds (HBs) are among the most prevalent and fundamental interactions occurring in nature. It is worth mentioning that they regulate various chemical (e.g., water association), physical (e.g., phase transitions), and biochemical (e.g., the formation of the DNA double helix responsible for transferring genetic information during replication) processes^1^. Furthermore, HBs play a significant role in molecular self-assembly mechanism by governing and stabilizing the structural organization of associating liquids (such as water^2^ and alcohols^3^ or more complex systems (i.e., liquid and plastic crystals^4,5^, polymers^6^, etc.). The issue of molecular self-organization is interesting but far from completely established and explained. For this reason, a great effort is put into intensive studies on this process, which is reflected in numerous theoretical and experimental articles^7–10^.

Since the formation and strength of HBs are susceptible to the variation in the external conditions, extensive investigations at various temperatures (*T*)^11^, elevated pressure (*p*)^12,13^, in a high electric field^14^, in nano/mesopores^15,16^, as well as in the presence of solvents^17,18^, are carried out to understand the nature of these specific interactions. Another interesting approach toward a better comprehension of HBs is the exchange of hydrogen by deuterium (H/D). Such a subtle substitution of the smallest possible molecular component may considerably impact the physical and thermodynamic properties of substances. A good illustration of this situation is the simple case of H_2_O and D_2_O, for which differences in thermodynamic and structural properties^19,20^, the melting/boiling point^21^, the temperature of the maximum density^22^ are reported and explained considering solely the variation between the mass of D and H nuclei. Furthermore, it should be mentioned about the isotope effects related to the geometry of the hydrogen bridge and its elongation due to the H/D replacement. This issue was raised, e.g., in Ref.^23^, where the authors indicated that the differences in the unit cell parameters between oxalic acid dihydrate and its deuterated derivative are probably associated with this phenomenon. Moreover, initially, for hydrocarbons, the variations in C–H versus C–D bond lengths were used to explain various molar volume effects due to the isotopic substitution^24^. However, further findings stated that differences in the intermolecular H∙∙∙H and D∙∙∙D distances are rather the controlling factors responsible for these effects^25,26^.

It is known that the isotopic substitution can also lead to the formation of new polymorphs. In this context, one can mention the crystallization studies of acridine and its deuterated derivative from solutions^27^. In acetone, in contrast to deuterated acridine, which transforms to form III, the native compound favors recrystallization to polymorphic form II. On the other hand, Mootz and Schilling^28^ revealed that the H/D exchange in the case of tetrahydrated trifluoroacetic acid leads to a transition from ionic (i.e., (H_5_O_2_)[CF_3_COO)_2_H]·6H_2_O phase) to molecular structure (i.e., per deuterated tetrahydrate CF_3_COOD·4D_2_O). Finally, it should be noted that some reports^29–31^ demonstrated that the H/D exchange might also have an impact on the pharmacokinetics and toxicity of drugs. On the example of the first deuterated drug for the treatment of Huntington’s disease-related movement disorders—deutetrabenazine^32^, it was shown that the presence of six D instead of H atoms in the structure causes the reduction of toxic metabolites, thereby lowering the occurrence of side effects^33^. However, it should be mentioned that there are also reports that show opposite effects caused by the deuteration^34,35^.

As far as there is an abundance of research about the effects of H/D replacement on the structure and properties of crystalline substances, only a few investigations are focused on the disordered—non-crystalline systems. Due to the strong development of research on the practical use of amorphous drugs, such studies would be extremely valuable. Therefore, in this paper, we present the results of Fourier transform infrared (FTIR) spectroscopy, differential scanning calorimetry (DSC), X-ray diffraction (XRD), as well as broadband dielectric spectroscopy (BDS) investigations carried out at ambient and elevated pressure for native melatonin, MLT, which is a class II drug according to the Biopharmaceutics Classification System^36^, and its partially-deuterated derivative (MLT-d_2_), in crystalline, supercooled and glassy phases. It should be stressed that previously, the impact of the isotopic exchange on the molecular dynamics of pharmaceutical glass-forming systems, especially at high-pressure conditions, which mimic those accompanying the tableting, has not been examined. In this context, it is worth emphasizing that the compaction of a substance into a tablet may subject it to stress and, consequently, initiate a phase transition (e.g., recrystallization, polymorphic transformation). As a result, the therapeutic effectiveness and safety of the active pharmaceutical ingredient (API) in the tablet may be changed. For these reasons, high-pressure dielectric studies are so important and should be performed on deuterated active substances. It is noteworthy that the results of our research together with outcomes of necessary pharmacological and pharmacokinetic investigations will allow bringing to the market new formulations containing amorphous deuterated APIs, characterized by improved properties when compared to their traditional (crystalline, non-deuterated) counterparts.

## Results and discussion

### FTIR data

As the first step of our studies, we carried out infrared measurements to characterize molecular vibrations in the native and partially-deuterated MLT and determine the degree of substitution of H by D in hydrogen bridges.

**Figure 1:**
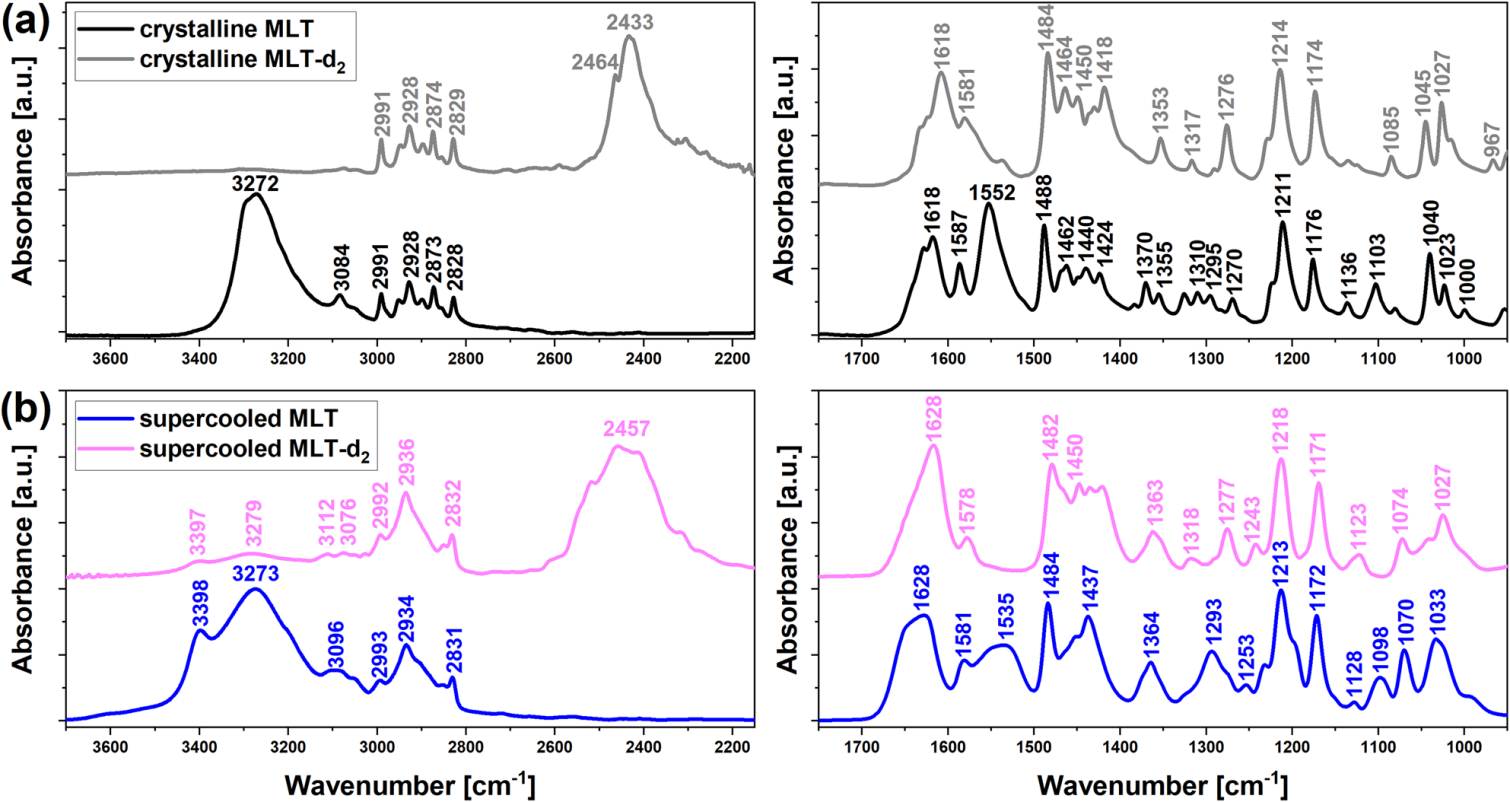
Infrared spectra of (**a**) crystalline MLT and MLT-d_2_ as well as (**b**) supercooled MLT and MLT-d_2_, measured at 295 K. The data are presented in two spectral regions: 3700–2150 cm^-1^ (left) and 1800–950 cm^−1^ (right).

**Figure 2:**
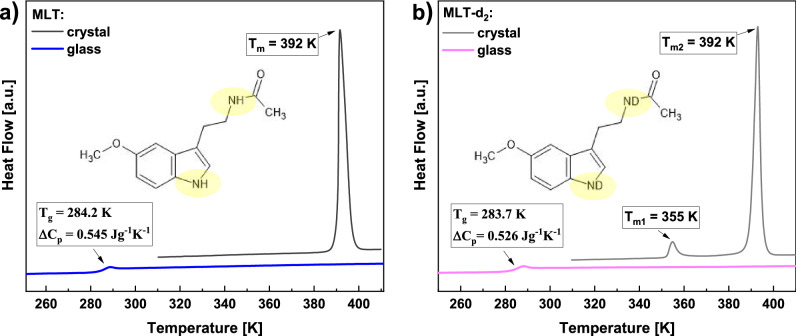
DSC thermograms obtained during heating of the crystalline and glassy MLT (**a**) and MLT-d_2_ (**b**) with the rate of 10 K/min. In the insets, the chemical structures of both substances are presented.

The representative FTIR spectra of MLT and MLT-d_2_, in the crystalline and supercooled liquid phases are presented in Fig. 1, while the chemical structures of these compounds are shown in Fig. 2. According to early XRD studies, the molecules of the crystalline MLT are connected in sheets by N–H···O hydrogen bonds with a N···O distance of 2.90 and 2.96 Å^37,38^. Here, the H-bonding interactions are reflected in the IR spectrum of crystalline MLT as a strong band with a maximum at 3272 cm^-1^ assigned to the stretching vibrations of two N–H groups. In the 3100–2800 cm^-1^ region, the bands related to different C-H stretching vibrations occur. Based on the calculated potential energy distribution (PED), for the minimum energy conformer of MLT, absorption bands at 2828, 2873, 2928, 2948, and 2991 cm^-1^ are assigned to symmetric and antisymmetric C-H stretching motions^39^. A signal occurring at 3084 cm^-1^ corresponds to the aromatic C-H stretching vibrations. In the 1700–900 cm^-1^ frequency range, the peaks associated with various vibrational modes, such as C=O, C–C, and C–N stretching, C-H, and N–H bending, are generally observed. There is also a signal at 1624 cm^-1^ due to the C=O stretching. According to Ref.^39^, a band occurring at 1587 cm^-1^ is attributed to the asymmetric C=C stretching and in-plane C–C–C bending of the benzene ring, whereas a signal at 1552 cm^-1^ corresponds to mixed asymmetric C=C stretching of the benzene ring and the C=C stretching of the five-membered ring. A peak occurring at 1488 cm^-1^ is related to the asymmetric C-N stretching of the amide group, in-plane bending of C–N–H moiety, and C-H scissoring of the methylene unit. Moreover, the bands located at 1462 and 1424 cm^-1^ are ascribed to C-H scissoring vibrations, whereas a peak at 1370 cm^-1^ corresponds to C-H symmetric deformation. The bands due to C-H twisting motions are observed at 1310 and 1295 cm^-1^, while those assigned to in-plane aromatic C-H bending vibrations occur at 1211 and 1136 cm^-1^. There are also peaks at 1176 cm^-1^ and 1040 cm^-1^, connected to C-O stretching vibrations and C-H rocking motion, respectively.

As shown in Fig. 1a,b, the IR spectra for the crystalline and supercooled MLT are similar, although the IR peaks for the latter sample are wider and blurred (no band splitting) compared to those collected for the crystalline API. This fact indicates a structural disordering in the supercooled MLT. In addition, the band at 3272 cm^-1^ attributed to the N–H stretching of amine groups is significantly broadened and gains an additional component with a frequency of 3398 cm^-1^, which denotes a change of hydrogen bonding interactions and a re-organization of the H-bond pattern after transformation of the sample from the crystalline to the disordered state.

For the crystalline MLT-d_2_, the peak from N–H vibrations at 3272 cm^-1^ is almost completely damped. On the other hand, the C-H bands for this compound exhibit nearly the same frequencies as for neat MLT. It indicates that the H/D exchange occurs only in N–H groups. As a result, the most significant changes in the spectral parameters are expected for the groups participating in the H-bond formation. Indeed, in the spectrum of MLT-d_2_, a new strong band at 2433 cm^-1^ assigned to the N–D stretching vibrations appears. From the integrated intensity ratio of the N–H to N-D bands, the degree of H/D substitution can be estimated. According to this method, the degree of H/D isotopic exchange is very high, > 99%, as almost no N–H absorption signal is observed in the 3500–3100 cm^-1^ frequency range. What is more, it was found that the partially-deuterated MLT exhibits extraordinary long-term stability since no reverse D/H exchange for over a dozen months of exposure to the air was noted. In addition, it is worth emphasizing that upon heating to the melting temperature and vitrification, only a slight loss of deuterons (~ 15%) was observed via D/H back-exchange with H atoms coming from the air. Thus, we demonstrated that it is possible to effectively convert MLT-d_2_ from the crystalline to the glassy state, without a significant loss of D atoms, by the vitrification of the molten system.

In the low-frequency spectral range, the bands at 1552, 1370, 1325, 1295, 1103, and 1000 cm^-1^, which were observed for neat crystalline MLT, disappeared for its deuterated derivative. On the other hand, a new peak of weaker intensity arose at 967 cm^-1^. Moreover, some of the bands for crystalline MLT-d_2_ in the range 1800–950 cm^-1^ were slightly shifted in relation to the corresponding peaks for MLT. The IR spectrum for supercooled MLT-d_2_ exhibited similar behavior as for the crystalline sample, i.e., the new band at 2457 cm^-1^ appeared (N–D stretching), while the signals at 1535, 1253, 1098 cm^-1^ disappeared compared to the native liquid MLT (Fig. 1b). The estimated amount of H/D-replaced sites in the N–H groups in the supercooled state was ca. 85%.

### DSC data

As a next step, we performed calorimetric measurements to characterize the thermal properties of the native and deuterated MLT. In Fig. 2a,b, DSC thermograms recorded on heating the crystalline and glassy forms of both substances are shown. In the case of initial (crystalline) samples, a well-visible endothermic process related to the melting at *T*_m_ = 392 K is observed. Moreover, in the thermogram of MLT-d_2_, there is an additional thermal event at *T* = 355 K. Further cooling followed by heating of the glassy MLT and its deuterated form revealed the presence of glass transition at nearly the same temperature (*T*_g_ = 284.2 and 283.7 K for MLT and MLT-d_2_, respectively). It should be noted that the obtained values of heat capacity jump at *T*_g_ ($\Delta$ *C*_p_) were also very similar for both substances ($\Delta$ *C*_p_ = 0.545 and 0.526 Jg^-1^ K^-1^ for MLT and MLT-d_2_, respectively).

To recognize the origin of the peculiar endothermic peak at *T* = 355 K for MLT-d_2_, especially verify whether it comes from a hydrate or H_2_O/D_2_O evaporation, thermogravimetric measurements were performed on the vacuum-dried deuterated and neat MLT recovered from the D_2_O/H_2_O solutions. As can be seen in Fig. S1 in the Supplementary Information (SI), there is a marginal loss of weight, within the experimental accuracy, upon heating the samples up to the decomposition temperature of both substances (*T* = 473 K). In fact, around *T* = 355 K, the weight loss curves look completely flat in deuterated and neat MLT. It suggests that the vacuum drying procedure applied in our experiments was effective and the samples are anhydrous. Note that a derivative analysis of the thermogravimetric data confirmed this conclusion. What is more, it was revealed that the weight-loss peak of MLT occurring at 598 K is shifted to 611 K for MLT-d_2_. It indicated that deuterated MLT exhibits greater thermal stability than the neat MLT. Additionally, it should be mentioned that the lack of hydrated water in both examined samples was also affirmed by the results of infrared investigations carried out on weakly (air) and hardly (vacuum) dried samples, as well as a comparison of the obtained IR data with those determined for selected crystalline hydrates (i.e., (3,4-dimethoxyphenyl)acetic acid monohydrate and bosentan monohydrate) and their anhydrous forms; for details see the SI (Figs. S2 and S3).

Hence, taking into account the results of the above experiments, we can conclude that the additional event visible in the thermogram of MLT-d_2_ at *T* = 355 K (Fig. 2) is not related to the evaporation of D_2_O/H_2_O released from the molten hydrates—the sample is anhydrous. Thus, one can suppose that this thermal effect is associated with some changes in the crystal structure. To verify the above hypothesis, we carried out XRD measurements.

### XRD data

**Figure 3:**
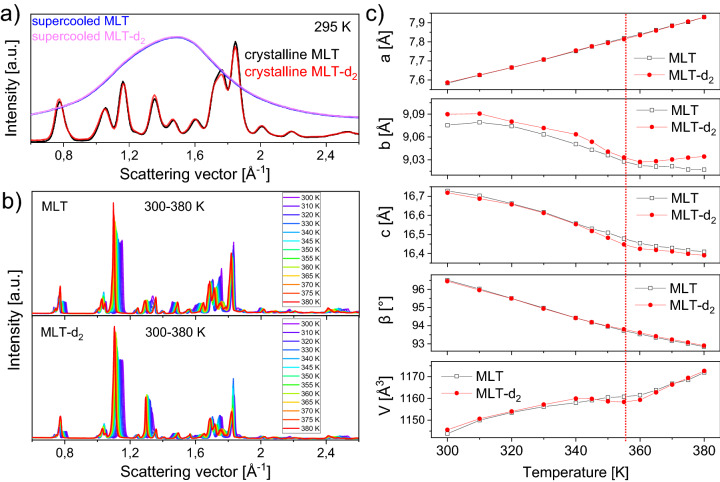
Comparison of XRD patterns, collected using Ag X-ray lamp, for MLT and MLT-d_2_ in the crystalline and supercooled liquid states at 295 K (**a**). The evolution of the diffraction patterns, collected using Cu X-ray lamp, during heating the initial crystals from 300 to 380 K (**b**). Panel (**c**) presents the changes in the unit cell parameters $a$, $b$,$c$, angle $\beta$, and volume $V$ as a function of temperature. The lines act as guides for the eye. The vertical dotted line highlights the point at *T* = 355 K for MLT-d_2,_ where the unit cell volume locally drops. Error bars are smaller than the plotted symbols.

Previous diffraction studies established that at 295 K MLT crystallizes in the space group *P*2_1_/*c* (Z = 4)^38^. The entire MLT molecule, except for hydrogen atoms, is approximately planar. The N–H···O HBs connect the MLT molecules in infinite sheets parallel to the $b$ and $c$ axes of the monoclinic unit cell. From the perspective of a supramolecular structure, the MLT molecule has one amide group and one indole group, and can act both as the acceptor and the donor of hydrogen bonds. Therefore, it is expected that MLT may form polymorphic forms when cocrystallizing with other compounds. The XRD patterns collected at room temperature using both Ag and Cu X-ray lamps on the commercial MLT powder (Fig. 3a and b) confirmed its crystal structure in the *P*2_1_/*c* space group. The refined unit cell parameters at 300 K are as follows: $a =$ 7.583(1) Å, $b =$ 9.075(1) Å, $c =$ 16.728(2) Å, $\beta =$ 96.508(4)°, $V =$ 1143.8(2) Å^3^. The parameters have slightly lower values than those determined for MLT monocrystal at 295 K by Quarles et al.^38^ ($a =$ 7.711 Å, $b =$ 9.282 Å, $c =$ 17.107 Å, $\beta =$ 96.77°). The diffractograms of freshly deuterated MLT-d_2_, measured at the same conditions, were set together in Fig. 3a and b. From this comparison, one can see that the crystal system of MLT-d_2_ resembles that of neat MLT. The diffraction peaks’ positions, widths, and intensities are very similar for both substances. The Pawley refinement of the diffraction pattern collected for MLT-d_2_ at 300 K using Cu radiation provided the following unit cell parameters: $a =$ 7.586(1) Å, $b =$ 9.090(1) Å, $c =$ 16.719(3) Å, $\beta =$ 96.454(6)°, $V =$ 1145.6(3) Å^3^. It means that the deuteration induced a slight non-isotropic unit cell distortion. The differences in the intensity ratios of subsequent diffraction peaks between the MLT and MLT-d_2_ systems at 295 and 300 K are also very subtle, and, therefore, no significant changes in the atomic organization are expected after the H/D exchange. Also, the structure of MLT and MLT-d_2_ in the supercooled liquid state is comparable since their XRD patterns (included in Fig. 3a) are almost identical. It indicates that the H/D substitution does not cause significant changes in the molecular arrangement of MLT-d_2_, in both crystalline and supercooled liquid phases.

The outcomes of the temperature-dependent XRD studies for crystalline MLT and MLT-d_2_ are shown in Fig. 3b. It can be noticed that with the increase in *T,* some peaks shift towards smaller, while the others towards higher scattering vectors (wider scattering angles), wherein the general crystal structure seems to be preserved and no polymorphic transformation is observed for both compounds. The revealed shifts in peak positions are due to an anisotropic thermal expansion of the unit cell. In order to quantitatively describe the evolution of the unit cell parameters with temperature, the Pawley method was applied to refine the structure. The results are shown in Fig. 3c and the examples of the refinements are shown in Fig. S4 in the SI. Based on this data, one can see that for both MLT and MLT-d_2_, $b$ and $c$ parameters exhibit a negative thermal expansion, while $a$ parameter increases rather linearly with *T.* The $\beta$ angle values also change linearly with *T.* Generally, for neat MLT, the variations in all unit cell parameters as a function of *T* are monotonic. However, for MLT-d_2_, one can see that there is a minimum in the $b$ dimension value at 360 K. Also, for the total unit cell volume, one can observe a minimum at around 355 K. Before and after this event, the trend in the unit cell volume changes for MLT-d_2_ remains virtually the same. Thus, around 355 K, a local negative thermal expansion of the MLT-d_2_ unit cell occurs. Although at this moment, we are not able to explain the origin of this effect, the fluctuation in the unit cell volume of MLT-d_2_ nicely corresponds to the additional thermal peak at 355 K revealed in the DSC thermogram. We suspect that it may be related to a subtle second-order phase transition of the order/disorder type. It is also worth noting that in literature, there are well-known examples where the H/D isotope exchange causes unexpected and poorly understood temperature variations of the unit cell parameters and molecular volume, e.g., in the case of deuterated polyethylene^26^ or H-, D- and halogen-substituted benzenes^40,41^. Since (*i*) one of the explanations of such effects is that they arise as a consequence of the different volume dependences of the internal vibrational frequencies between the hydrogenated and deuterated systems^26^, and (*ii*) one possible mechanism for the negative thermal expansion is associated with the soft-phonon process as a result of unusual lattice vibrations^42^, we can assume that the origin of the observed thermal effects may be related to changes in the lattice vibrations after D_2_O treatment.

After careful examination of the changes in the diffraction peak intensities as a function of temperature, one can notice that some of the Bragg reflections rise with the increase of this parameter. Typically, one expects that the intensity of the diffraction maxima from crystalline solid decreases with increasing temperature due to the thermal vibration of atoms. Here, most of the Bragg peaks for MLT as well as MLT-d_2_ rise with increasing *T*. However, the magnitude of these changes is stronger for MLT-d_2_. Therefore, one can suppose that the studied MLT systems experience a mild solid–solid phase transition at *T* below their melting points, which is related to the H or D arrangement. From the observed changes in the Bragg peak positions and intensities, the transitions do not appear sharp, as might be expected for a first-order transition. Further studies are needed to better understand the origin of the observed temperature-dependent variations in the structure of MLT and MLT-d_2_.

### BDS data

**Figure 4:**
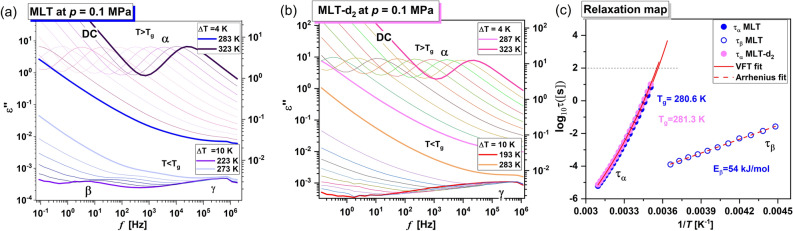
Dielectric loss spectra measured for MLT (**a**) and MLT-d_2_ (**b**) at ambient pressure, in a wide temperature range, above and below the *T*_g_. Panel (**c**) presents a relaxation map for both examined compounds. Solid and dashed red lines are VFT and Arrhenius fits, respectively.

After XRD studies, we have carried out ambient pressure (*p* = 0.1 MPa) BDS measurements on the glassy/supercooled MLT and MLT-d_2_. As demonstrated in Fig. 4a,b, dielectric loss spectra were collected at temperatures both above and below the glass transition temperature, *T*_g_. At *T* > *T*_g_, two specific processes can be recognized in the spectra of the examined substances. The first one (marked as DC, i.e., dc-conductivity) is associated with the charge transport of some residual ionic impurities, while the second one located at higher frequencies (*f*) and called the structural (*α*)*-*relaxation, is related to the cooperative motions of molecules. It is well-seen that both processes shift towards lower *f* with decreasing *T*.

On the other hand, the loss spectra measured at lower *T* (below the *T*_g_) reveal a presence of one (in MLT-d_2_) or two (in MLT) secondary relaxation processes. The faster *γ*-mode is visible in the spectra of both substances. In turn, the slower *β*-process with a small amplitude is detected only in the neat MLT.

To accurately characterize the molecular dynamics of the investigated systems at *p* = 0.1 MPa and in a wide *T* range, we have used the Havriliak-Negami (HN) function. The formula for fitting the measured dielectric loss spectra has the following form^43^:

1 $$\varepsilon \left( \varpi \right)^{^{\prime\prime}} = \frac{{\sigma_{dc} }}{{\varepsilon_{0} \varpi }} + {\text{Im}} \mathop \sum \limits_{i = 1}^{2} \left( {\varepsilon_{\infty } + \frac{{\Delta \varepsilon_{i} }}{{[1 + (i\varpi \tau_{i} )^{{\alpha_{i} }} ]^{{\beta_{i} }} }}} \right),$$

where $\sigma_{dc}$ is the dc-conductivity, *ε*_0_ is the vacuum permittivity, $\overline{\omega }$ is an angular frequency ($\overline{\omega }$ = 2π*f*), $\varepsilon_{\infty }$ is the high-frequency limit permittivity, Δ*ε* is the dielectric relaxation strength, $\tau$ is the HN relaxation times, *α* and *β* are the shape parameters representing the breadth and asymmetry of given relaxation peaks. Next, to describe the temperature dependencies of *τ*_*α*_ (which were previously recalculated from $\tau$ using the expression given in Ref ^44^ for both systems (Fig. 4c), the Vogel–Fulcher–Tammann (VFT) equation was used^45–47^:

2 $$\tau_{\alpha } = \tau_{VFT} \exp \left( {\frac{{D_{T} T_{0} }}{{T - T_{0} }}} \right),$$

where $\tau_{VFT}$ is the time scale of vibrational movements, $D_{T}$ is the strength parameter, and $T_{0}$ represents the temperature at which structural times tend to infinity. Based on VFT fits, we have estimated *T*_g_ (defined as *T* at which $\tau_{\alpha }$ = 100 s) at *p* = 0.1 MPa for the native and partially-deuterated API. It should be noted that the obtained values (*T*_g_ = 280.6 and 281.3 K for MLT and MLT-d_2_, respectively) are very similar to those estimated from the DSC technique (*T*_g_ = 284.2 and 283.7 K, respectively, see Fig. 2).

In the case of analysis of the secondary modes (*β* and *γ*), obtaining reliable values of relaxation times from the fitting using Eq. 1 was possible exclusively for the *β*-process in MLT. For the *γ-*mode, it was not feasible since the maximum of this peak did not enter the experimental frequency window. From the further analysis of the dependence log_10_*τ*_*β*_ vs. 1/*T* with applying the Arrhenius equation (dashed red lines in Fig. 4c):

3 $$\tau_{\beta } = \tau_{\infty } \exp \left( {\frac{{E_{\beta } }}{RT}} \right),$$

where $\tau_{\infty }$ is a pre-exponential factor, and *R* is a gas constant, the activation energy for this relaxation (*E*_*β*_ (MLT) = 54 kJ/mol) was determined.

**Figure 5:**
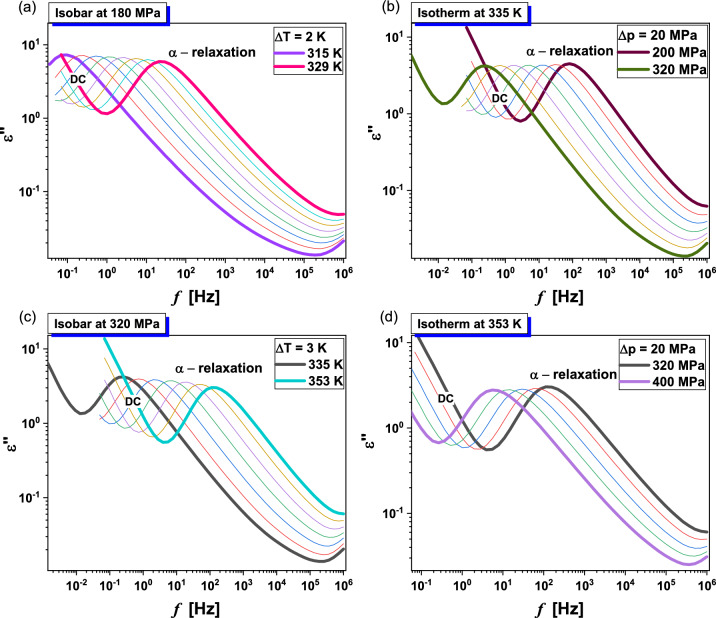
Representative dielectric loss spectra measured for MLT at isobaric [panels (**a**) and (**c**)] and isothermal [panels (**b**) and (**d**)] conditions.

**Figure 6:**
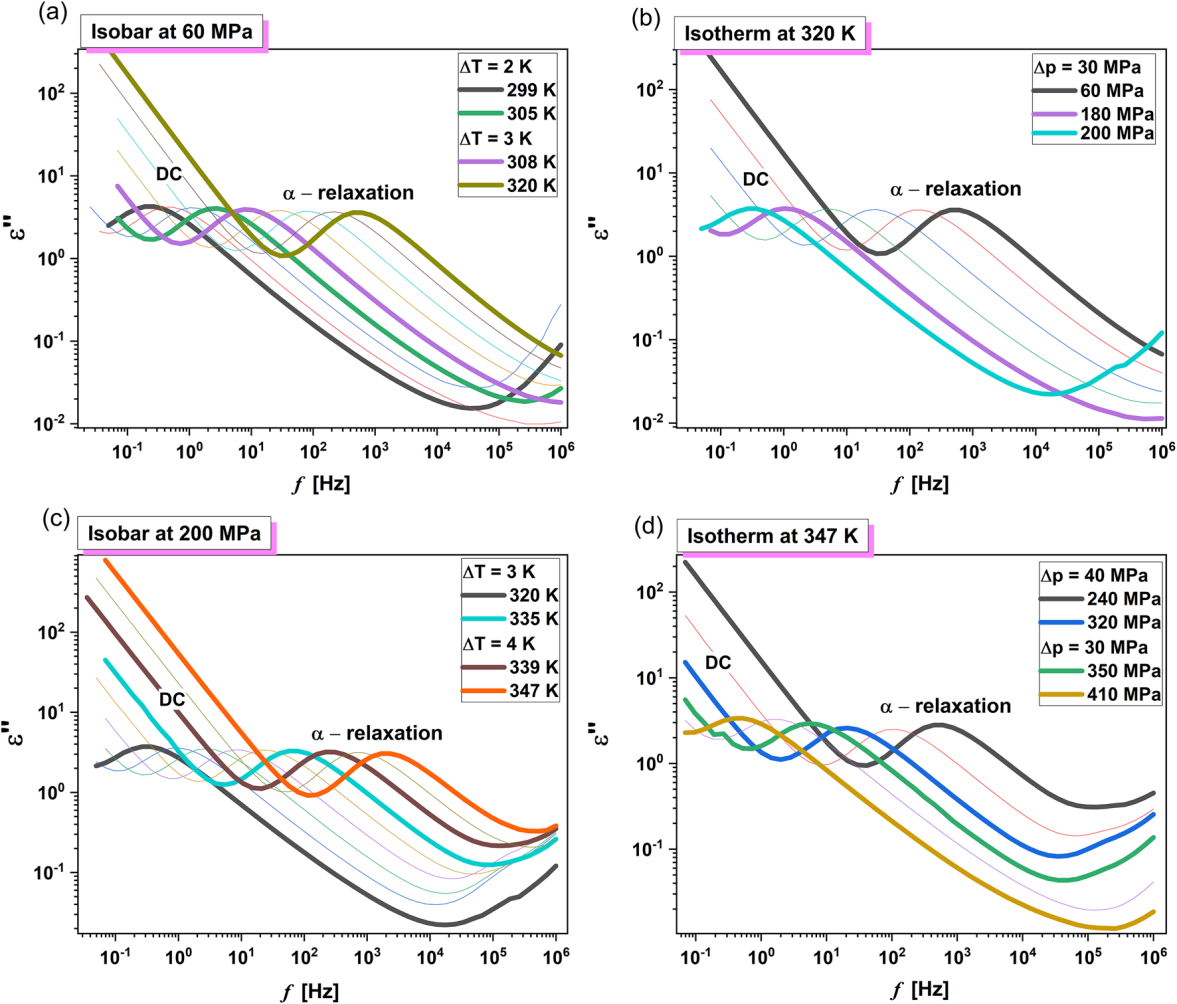
Representative dielectric loss spectra measured for MLT-d_2_ at isobaric [panels (**a**) and (**c**)] and isothermal [panels (**b**) and (**d**)] conditions.

Subsequently, we performed extensive high-pressure dielectric investigations at isobaric and isothermal conditions. In Figs. 5 and 6, representative dielectric loss spectra collected for MLT and MLT-d_2_ at constant *p* and various *T* higher than *T*_g_ (panels a,c), as well as at constant *T* and indicated *p* < *p*_g_, (panels b,d) are presented. As in the case of ambient pressure studies, except for the DC, a structural (*α*)-relaxation peak is observed in the spectra of both examined substances. Its maximum moves towards lower *f* with decreasing temperature or increasing pressure.

**Figure 7:**
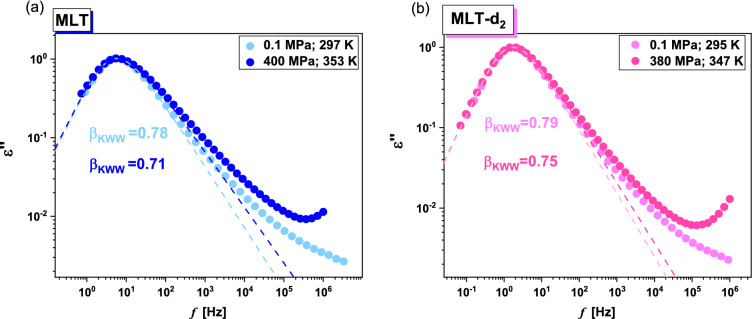
A comparison of dielectric spectra obtained at different thermodynamic conditions (*T*, *p*), close to *T*_g_ for MLT (**a**) and MLT-d_2_ (**b**). They were normalized with respect to the maximum of dielectric loss (*ε*”_max_). The dashed lines represent KWW fits.

In Fig. 7, the normalized dielectric spectra for MLT and MLT-d_2_ measured at ambient and very high *p*, close to *T*_g_ (the maxima of *α*-peaks near 10 Hz) were compared. As can be seen, for both substances, the *α*-dispersion broadens with compression. It means the breakdown of the fundamental rule, called the Temperature Pressure Superpositioning (TPS), saying that under various *T* and *p* conditions, for a constant *τ*_*α*_, the width of the structural peak becomes unchanged^48^. This effect is more pronounced in the case of neat MLT. To visualize it, the presented spectra were analyzed by means of the one-sided Fourier transform of the Kohlrausch–Williams–Watts (KWW) function (dashed lines)^49,50^:

4 $$\Phi_{KWW} \left( t \right) = \exp [ - \left( {t/\tau_{\alpha } )^{{\beta_{KWW} }} } \right].$$

It was found that the difference between the value of the stretched exponent ($\beta_{KWW}$) obtained from the fitting of the spectra collected at 400 and 0.1 MPa (MLT), as well as 380 and 0.1 MPa (MLT-d_2_), was equal to 0.07 and 0.04, respectively. It should be noted that the broadening of the *α*-peak with increasing *p* (violation of the TPS rule) is typical for associating liquids, mainly those creating complex H-bonded networks, e.g., m-fluoroaniline^51^, or polyalcohols^52^. According to the literature data, the reason for this is the variation in the populations of HBs at different *T* and *p*, resulting in enhanced intermolecular coupling between the molecules. MLT as an associating system shows similar behavior. Nevertheless, it is clearly visible that the impact of pressure on the shape of the structural process in the native and partially-deuterated MLT is not the same. Such experimental observation is a direct manifestation of the variation in dynamical as well as static properties of hydrogen (N–H⋅⋅⋅O) and deuterium (N–D⋅⋅⋅O) bonds at various thermodynamic conditions.

**Figure 8:**
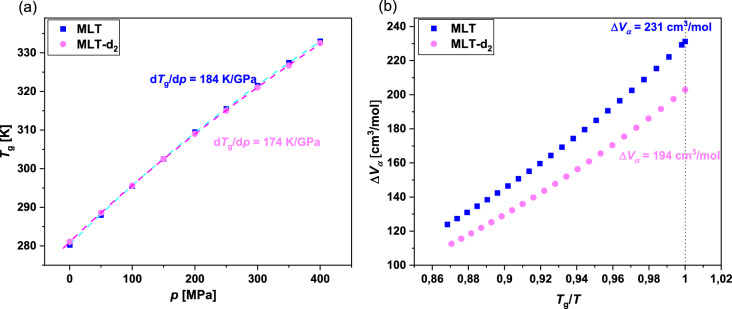
*T*_g_ plotted as a function of *p* for MLT and MLT-d_2_ (**a**). The solid lines present the fits using Eq. 5. Dependence of the activation volume for the *α*-process (Δ*V*_*α*_) versus *T*_g_/*T* for both substances (**b**).

In the next step, dielectric spectra collected for MLT and MLT-d_2_ at elevated *p* (Figs. 5 and 6) were analyzed using the single HN function with the conductivity term [Eq. (1)]. Then, we plotted the obtained structural relaxation times, $\tau_{\alpha }$ (firstly recalculated from $\tau$, see Ref.^44^) as a function of two variables: *T* and *p* in Fig. S5a,b in the SI. These dependencies, which formed two-dimensional surfaces within the range of investigated temperatures and pressures, were subsequently fitted to the modified Avramov expression (Equation S1). By utilizing the Avramov fits (parameters collected in Table S1 in the SI), two quantities, which are indicators of the sensitivity of the structural relaxation to compression, i.e., the pressure coefficient of the glass transition temperature (d*T*_g_/d*p*) and activation volume for the *α*-process (Δ*V*_*α*_), have been estimated. To obtain the first one, we calculated the values of $T_{g}$ using the following formula proposed by Avramov^53^:

5 $$T_{g} \left( p \right) = T_{g} \left( {p_{0} } \right)\left( {1 + \frac{p}{\Pi } } \right)^{{\beta /\alpha_{0} \left( {1 - \frac{C}{{C_{{p_{0} }} }} \left( {\ln \left( {1 + \frac{p}{\Pi }} \right)} \right)} \right)}}$$

with the same parameters (${\Pi }$, $\beta$, $\alpha_{0}$, and $C_{{p_{0} }}$) as those occurring in equation S1 (see Table S1 in the SI), and next presented them versus *p* in Fig. 8a. From this plot, we determined d*T*_g_/d*p* in the limit of ambient *p* for MLT (184 K/GPa) and its deuterated derivative (174 K/GPa).

Additionally, Δ*V*_*α*_ for both considered compounds was estimated at various *T* by applying the definition (equation S4 in the SI) directly from the modified Avramov model. The dependencies of Δ*V*_*α*_* vs*. *T*_g_/*T* are presented in Fig. 8b. The values of Δ*V*_*α*_ calculated for MLT and MLT-d_2_ at *T*_g_ (*T*_g_/*T* = 1) were equal to 231 and 194 cm^3^/mol, respectively.

Analyzing the d*T*_g_/d*p* and Δ*V*_*α*_ (at *T*_g_) parameters for examined associating substances, it can be concluded that the *α*-process in MLT is a bit more affected by pressure (density variation) than in its partially-deuterated form. Generally, the obtained values are quite high, which means a strong impact of compression on the structural dynamics in both systems. Herein, it is worth reminding that for typical but strongly H-bonded systems, such as polyalcohols, rather lower d*T*_g_/d*p* (< 70 K/GPa)^54^, and Δ*V*_*α*_ at *T*_g_ (< 40 cm^3^/mol)^55^ have been obtained. However, it should be mentioned that similar, in comparison to MLT, values of the first parameter, i.e., d*T*_g_/d*p* coefficient, have been reported for several weakly H-bonded active substances, e.g., fluconazole (183 K/GPa)^56^, ibuprofen (195 K/GPa)^57^, salol (204 K/GPa)^58^, or curcumin (197 K/GPa)^59^. Note that also in Refs.^56,58^, a slightly greater or lower Δ*V*_*α*_ with respect to MLT has been given for fluconazole and salol (Δ*V*_*α*_ = 296 and 170 cm^3^/mol, respectively). The difference in the pressure coefficient of the glass transition temperature, and especially activation volume at the *T*_g_ for both compounds, clearly show that the dynamics of HBs in the native and partially-deuterated MLT is different and influences the structural relaxation. From the analysis of both parameters, it becomes clear that the densification process has a larger impact on the dynamics in the former system, which might indicate (it is just a hypothesis) that along with the change in the strength of HBs, also the variation in the architecture of associates occurs in MLT-d_2_. This supposition is made considering the value of the activation volume calculated in the limit of ambient pressure at varying temperatures that changed disproportionally strong with respect to the molecule volume due to the replacement of H by D.

## Conclusions

In this paper, XRD, FTIR, DSC, and BDS techniques have been applied to study the atomic and molecular structure, hydrogen bonds, thermal properties, and molecular dynamics at ambient and elevated pressure in melatonin and its partially-deuterated derivative. IR spectroscopy investigations showed that the H/D substitution in MLT molecules occurs only in the amine N–H groups and the degree of H/D exchange is very high (above 95% of H atoms replaced by D). Moreover, they revealed excellent stability of the obtained deuterated derivative of the examined substance, even after a dozen months of exposure to the air as well as after melting and subsequent vitrification. Further calorimetric measurements indicated the presence of an additional endothermic event for MLT-d_2_ at ~ 355 K compared to neat MLT, where only melting and glass transition were recorded. XRD studies demonstrated that both MLT and MLT-d_2_ exhibit an anisotropic thermal expansion of the monoclinic unit cell, where the lattice parameter $a$ extends, while the $b$ and $c$ parameters, as well as $\beta$ angle, generally decrease with the increase in temperature. However, for only MLT-d_2_, there is a fluctuation in these parameters at ~ 355 K, resulting in the local negative thermal expansion of the unit cell volume. This subtle structural transition may explain the peculiar thermal peak revealed in the DSC curve. MLT can be thus classified as compound exhibiting unusual, non-monotonic thermal expansion changes of the unit cell volume as a result of the D_2_O treatment. Furthermore, dielectric investigations performed at different *T* and *p* conditions showed that: (*i*) the TPS rule is not fulfilled in both native and partially-deuterated MLT, (*ii*) there is a variation in the pressure dependence of the glass transition temperature—consequently, slightly different, however relatively high values of d*T*_g_/d*p* parameters (184 and 174 K/GPa for MLT and MLT-d_2_, respectively), indicating substantial sensitivity of the *α*-relaxation to compression, were determined for both substances, (*iii*) the temperature evolution of Δ*V*_*α*_ (and hence, the calculated Δ*V*_*α*_ at *T*_g_) clearly varies for MLT and MLT-d_2_. Based on the above results, we concluded that (*i*) there are fluctuations in the dynamical and static properties of hydrogen and deuterium bonds for both examined compounds at different thermodynamic conditions, and (*ii*) the dynamics of MLT is more affected by the densification process in comparison to MLT-d_2_. These are quite interesting findings worthy of more detailed studies in the future. It should also be mentioned that the results presented in this paper are a preliminary stage for further, very important research, including solubility studies in various pH and biorelevant media. Moreover, pharmacological and pharmacokinetic investigations to establish whether the formation of partially-deuterated MLT does not lead to undesired effects need to be carried out as well. Importantly, the collection of the outcomes of all the above-mentioned research can contribute to the introduction to the market of amorphous deuterated APIs in the near future.

## Methods

### Materials

Neat MLT (*M*_*w*_ = 232.3 g/mol) with purity greater than 98% was purchased from Sigma Aldrich and used as received. MLT-d_2_ (*M*_*w*_ = 234.3 g/mol) has been prepared by dissolution of a drug (200 mg) in deuterium oxide (D_2_O, 25 ml). The solution was evaporated on the Rotavapor® R-100 (Büchi, Flawil, Switzerland). The flask filled with the solution was immersed in the heating bath at 80 °C and rotated at 140 rpm. The internal pressure was initially set at 400 mbar and then reduced to 10 mbar step-wisely.

### FTIR

FTIR spectra were recorded using a Thermo Scientific Nicolet iS50 spectrometer equipped with a DTGS detector, KBr beam splitter, and a built-in diamond attenuated total reflection (ATR). Each sample was placed in the center of the diamond prism and immediately measured. Spectra were collected at 295 K in the wavenumber range 400‒4000 cm^–1^ with a resolution of 4 cm^–1^ (32 scans).

### DSC

Calorimetric measurements were carried out using a Mettler-Toledo DSC apparatus equipped with a liquid nitrogen cooling accessory and an HSS8 ceramic heat flux sensor with 120 thermocouples. The instrument was calibrated for temperature and enthalpy using indium and zinc standards, while heat capacity *C*_p_ calibration was done using a sapphire disc. Investigated compounds (crystalline MLT, MLT-d_2_) were placed in aluminum crucibles (40 µL) and sealed. Next, they were heated above their melting temperature, immediately cooled to vitrify the liquid substances, and after that, scanned over a *T* range from 250 K to well above the glass transition point (up to 410 K). The heating/cooling rate during the experiments was equal to 10 K/min.

### XRD

The diffraction measurements for the crystalline and supercooled MLT and MLT-d_2_ phases at room temperature (295 K) were performed using a Rigaku-Denki D/MAX RAPID II-R diffractometer equipped with a rotating silver anode, an incident beam graphite monochromator (Ag Kα_1,2_, wavelength ${\uplambda }$ = 0.5608 Å), and an image-plate as a two-dimensional detector in the Debye–Scherrer geometry. The supercooled samples were prepared by melting the crystalline samples at 385 K and cooling down to 295 K. The *T*-dependent studies of the crystalline phases were performed with a Malvern Panalytical Empyrean diffractometer using a nickel-filtered Cu Kα_1,2_ source (λ = 1.5406 Å) and equipped with a PIXcell^3D^ ultra-fast solid-state hybrid detector. This set-up provides better angular resolution than the former diffractometer and, therefore, was used to analyze the unit cell parameters. An Anton Paar TTK 450 temperature chamber was applied for temperature control. Measurements were carried out at the range of 300‒380 K, in a reflection mode, in the Bragg–Brentano geometry (θ–θ scan technique), within the 2θ range of 10–40°. All diffraction data were presented as a function of the scattering vector ${\text{Q}} = 4\uppi \sin\uptheta /\uplambda$. The Pawley refinement was performed using the FullProf program suite^60^ and the unit cell parameter dependencies were determined as a function of *T* for both compounds.

### BDS

Ambient and elevated pressure measurements of the complex dielectric permittivity, ε*(ω) = *ε*′(ω) − i*ε*″(*ω*), over a frequency range of 10^−3^‒10^6^ Hz, were taken using a Novo-Control Alpha dielectric spectrometer. The temperature control (at 0.1 MPa) was provided by Quatro System using a nitrogen gas cryostat, with stability better than 0.1 K. The samples were placed between two stainless-steel electrodes, separated by a thin Teflon spacer (diameter, 20 mm; gap, 0.075 mm). For dielectric experiments at elevated compression, a high-pressure chamber was used. The sample capacitor was covered thoroughly by Teflon tape, which ensured separation from the silicone oil. The pressure was measured using a Nova Swiss tensometric meter with a resolution of 1 MPa. Moreover, we applied a refrigerated and heating Huber circulator to regulate the temperature with a precision of 0.1 K.

Isothermal and isobaric measurements for both examined compounds at temperatures above the glass transition temperature (*T*_g_) and below the glass transition pressure (*p*_g_) were performed in the following thermodynamic conditions:

isothermal measurements: *T* = 313 K (*p* = 0.1 − 180 MPa), *T* = 335 K (*p* = 200 − 320 MPa) and *T* = 353 K (*p* = 320 − 400 MPa) for MLT; *T* = 299 K (*p* = 0.1 − 60 MPa), *T* = 320 K (*p* = 60 − 200 MPa) and = 347 K (*p* = 240 − 410 MPa) for MLT-d_2_.

isobaric measurements: *p* = 180 MPa (*T* = 313 − 335 K), and *p* = 320 MPa (*T* = 335 − 353 K) for MLT; *p* = 60 MPa (*T* = 299 − 320 K), *p* = 200 MPa (*T* = 320 − 347 K) for MLT-d_2_.

## Supplementary Information

Supplementary Information.

## Data Availability

The datasets used and/or analyzed during the current study available from the corresponding author on reasonable request.
