# Supplementary material for: The impact of H/D exchange on the thermal and structural properties as well as high-pressure relaxation dynamics of melatonin
Source: Sci Rep. 2022 Aug 22;12:14324. doi: 10.1038/s41598-022-18478-0 (PMC9395371; doi:10.1038/s41598-022-18478-0)
Supplement: Supplementary file 1 — Supplementary Information. [file 41598_2022_18478_MOESM1_ESM.docx]

**The impact of H/D exchange on the thermal and structural properties as well as high-pressure relaxation dynamics of melatonin**

Paulina Jesionek^1,2^, Barbara Hachuła^1*^, Dawid Heczko^2^, Karolina Jurkiewicz^3*^, Magdalena Tarnacka^3^, Maciej Zubko^4,5^, Marian Paluch^3^, Kamil Kamiński^3^, Ewa Kamińska^2^

^1^ *Institute of Chemistry, Faculty of Science and Technology, University of Silesia in Katowice, 40-007 Katowice, Poland*

^2^ *Department of Pharmacognosy and Phytochemistry, Faculty of Pharmaceutical Sciences in Sosnowiec, Medical University of Silesia in Katowice, 41-200 Sosnowiec, Poland*

^3^ *Institute of Physics, Faculty of Science and Technology, University of Silesia in Katowice, 41-500 Chorzow, Poland*

^4^ *Institute of Materials Engineering, Faculty of Science and Technology, University of Silesia in Katowice, 41-500 Chorzow, Poland*

^5^ *Department of Physics, Faculty of Science, University of Hradec Králové, 500 03 Hradec Králové, Czech Republic*

SUPPLEMENTARY INFORMATION

**Thermogravimetric data**

Water evaporation from MLT (dried from H_2_0) and MLT-d_2_ was examined by a Mettler TG 50 thermogravimetric analyzer linked to a Mettler MT5 balance (Mettler Toledo, Switzerland). The powder in open aluminum pans was placed in a furnace under nitrogen purge (30 mL min^−1^) and heated at 10 K min^−1^ from room temperature to *T*=1150 K. Water evaporation of the sample was determined by the weight loss percentage.

**Figure S1.** TGA-DTG thermograms of MLT (dried from H_2_O) and MLT-d_2_.

**FTIR data**

**Figure S2.** FTIR spectra of anhydrous and hydrous crystalline MLT and MLT-d_2_ presented in

two spectral regions: 3750-2000 cm^-1^ (left) and 1750-400 cm^-1^ (right).

Comparing the spectra presented in Fig. S2, it is well visible that the samples containing water (hydrated) have a broad band in the range of 3700‒3350 cm^-1^ corresponding to the stretching vibrations of the O-H groups of water molecules, while the anhydrous samples (dried under vacuum) do not. Thus, one can state that MLT and MLT-d_2_ samples obtained after vacuum drying are anhydrous.

Furthermore, in Fig. S3, we present the FTIR spectra of selected crystalline hydrates (i.e., (3,4-dimethoxyphenyl)acetic acid monohydrate and bosentan monohydrate) and their anhydrous forms, to demonstrate the possible positions of bands originating from the O-H stretching vibrations of hydrated water molecules (highlighted in yellow).^[[1]](#endnote-1),^^[[2]](#endnote-2)^ Based on these data, it is definite that MLT and MLT-d_2_ samples do not contain water in their crystal structure (no O-H bands with a subtle structure occur in the 3600‒3400 cm^-1^ range in their FTIR spectra).

**Figure S3.** FTIR spectra of anhydrous and hydrated (3,4-dimethoxyphenyl)acetic acid (34DMPA) and bosentan (BOS) presented in the regions of 3800-2200 cm^-1^ (left) and 1800-400 cm^-1^ (right). The bands assigned to the stretching vibrations of O-H groups (from water molecules) are highlighted in yellow.

**X-ray diffraction data**


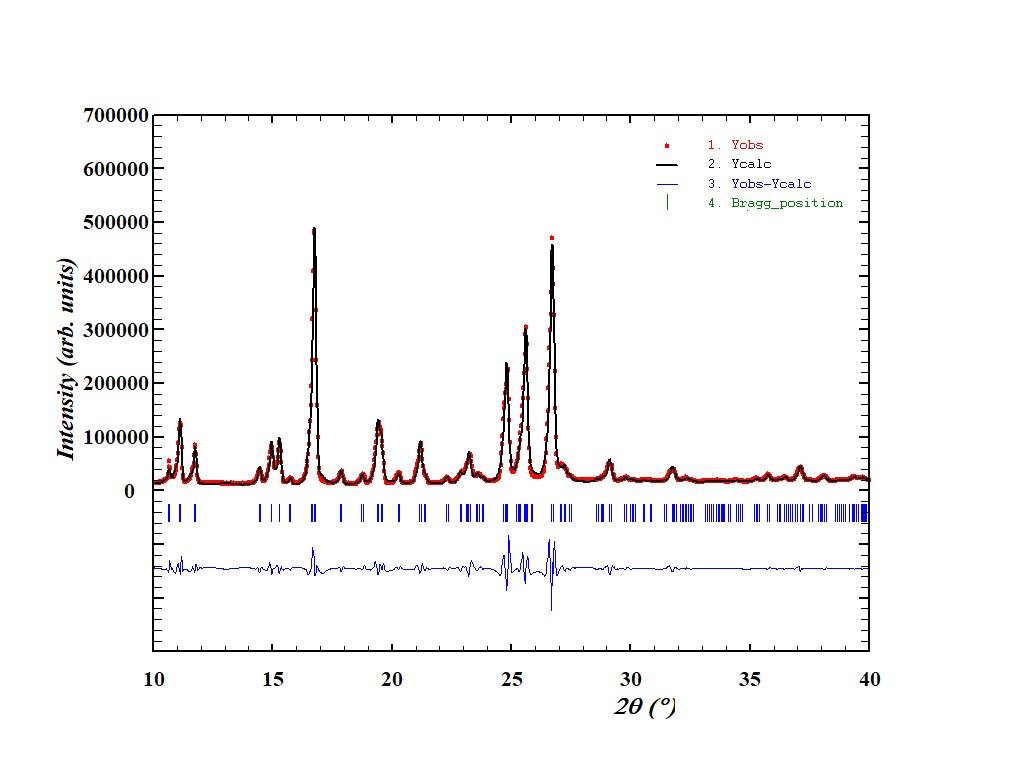

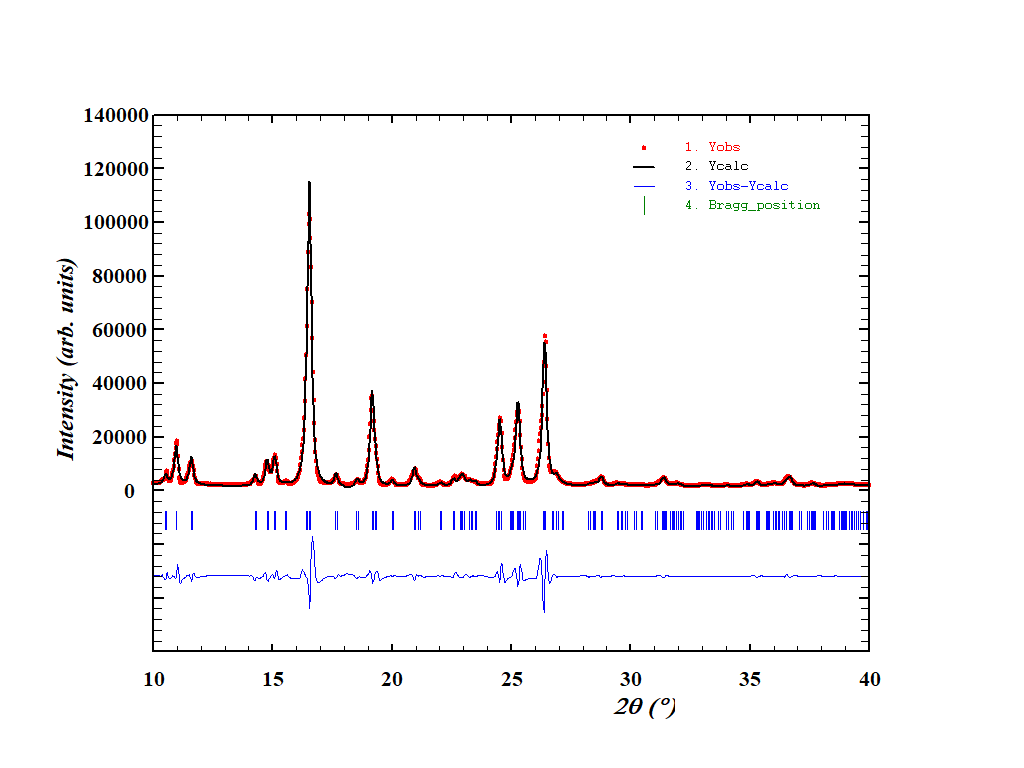


| 4. Bragg_position

| 4. Bragg_position

**Figure S4.** Examples of the Pawley refinement of the diffraction data for MLT (left) and
MLT-d_2_ (right) at 300 K.

**Dielectric data**

**Figure S5.** Structural (*α*)-relaxation times plotted as a function of *T* and *p* for MLT (a) and MLT-d_2_ (b). Blue and pink areas represent surface fits to equation S1.

**Table S1.** Parameters of the modified Avramov equation (equation S1) obtained from the analysis of *τ_α_* (*T*,*p*) dependencies for MLT and MLT-d_2_.

|  | **MLT** | **MLT-d_2_** |
| --- | --- | --- |
| log_10_(*τ_∞_*[s]) | -11.15±0.30 | -11.15±0.33 |
| *T_r_* [K] | 280.3±0.1 | 281.1±0.1 |
| *α*_0_ | 5.603±0.195 | 5.601±0.211 |
| *C/C_p_*_0_ | 0.048±0.015 | 0.087±0.017 |
| Π [MPa] | 435.8±24.4 | 441.7±24.8 |
| *β* | 1.435±0.067 | 1.376±0.083 |
| Adj. R-Square | 0.999 | 0.999 |

The data shown in Fig. S5 were analyzed using the Avramov equation^[[3]](#endnote-3)^:

$\tau_{\alpha}=\tau_{\infty}exp\left[ {\ln{(\tau}_{g}/\tau_{\infty})\left( \frac{T_{r}}{T} \right)}^{\alpha_{0}\left( 1-\frac{C}{C_{p_{0}}}\ln\left( 1+\frac{p}{\Pi} \right) \right)}\left( 1+\frac{p}{\Pi} \right)^{\beta} \right]$, (S1)

where $\tau_{\infty}$ is a relaxation time at extremely high *T*, $\tau_{g}$=$\tau(T_{g})$, $T_{r}$ is a reference temperature lying close to the$T_{g}$, $C_{p_{0}}$ is a specific heat capacity, $C$ is an additional adjustable parameter, $\Pi$ is a constant with the dimension of pressure, $\alpha_{0}$ and $\beta$ are exponential parameters, which are linked to the thermodynamic quantities via the following relations:

$\alpha_{0}=\frac{2C_{p_{0}}}{ZR}$, (S2)

$\beta=\frac{2\alpha_{p}V_{m}}{ZR}\Pi$**,** (S3) where *Z* represents the degeneracy of the system,$\alpha_{p}$ is a volume expansion coefficient at ambient *p*, and $V_{m}$ is a molar volume. In Table S1, the parameters of equation S1 determined from the global numerical fitting (Fig. S5), are presented.

By applying the following definition:

$\Delta V_{\alpha}=RTln10{(\frac{d log\tau_{\alpha}}{dp})}_{T}$ (S4)

we calculated the activation volume for the *α*-process ($\Delta V_{\alpha}$) directly from the modified Avramov model, see Fig. 8b.

**References**

1. . Hachuła, B., Nowak, M. & Kusz, J. Hydrogen-bonding interactions in (3,4-dimethoxyphenyl) acetic acid monohydrate. *Acta Cryst. C* **64**, o357–o360 (2008). [↑](#endnote-ref-1)
2. . Minecka, A. *et al.* Studies on the vitrified and cryomilled bosentan. *Mol. Pharm.* **19**, 80–90 (2022). [↑](#endnote-ref-2)
3. . Avramov, I. Pressure dependence of viscosity of glass-forming melts. *J. Non-Cryst. Solids* **262**, 258–262 (2000**)**. [↑](#endnote-ref-3)
